# 超高效液相色谱-串联质谱法测定化妆品中新型糖皮质激素氯倍他索乙酸酯

**DOI:** 10.3724/SP.J.1123.2022.06010

**Published:** 2023-03-08

**Authors:** Piaopiao YANG, Wei HUANG, Lixia LI, Hong LIU

**Affiliations:** 湖北省药品监督检验研究院, 湖北省药品质量检测与控制工程技术研究中心, 湖北 武汉 430075; Hubei Institute for Drug Control, Hubei Engineering Research Center for Drug Quality Control, Wuhan 430075, China

**Keywords:** 超高效液相色谱-串联质谱, 糖皮质激素, 氯倍他索乙酸酯, 化妆品, ultra performance liquid chromatography-tandem mass spectrometry (UPLC-MS/MS), glucocorticoids, clobetasol acetate, cosmetics

## Abstract

采用超高效液相色谱-串联质谱法建立了化妆品中氯倍他索乙酸酯的检测方法,适用于膏霜乳类、凝胶类、泥类、贴膜类和液体(水)类5种常见化妆品基质。对影响被测物质的前处理方法、提取溶剂、提取时间等因素和色谱、质谱条件等进行了考察。最终建立方法为样品经乙腈涡旋分散、超声提取、过滤后用超高效液相色谱-串联质谱仪测定,采用Waters CORTECS C_18_色谱柱(150 mm×2.1 mm, 2.7 μm),以水和乙腈作为流动相分离,在电喷雾正离子模式(ESI^+^)下,以多反应监测(MRM)方式采集,采用基质匹配标准曲线进行定量分析。5种基质类型下,被测物质在0.9~37 μg/L的范围内线性拟合良好,线性相关系数(*R*^2^)均超过0.99。方法的检出限为0.03 μg/g,定量限为0.09 μg/g。在定量限、2倍定量限和10倍定量限3个水平下的加标回收率试验中,被测物质在5种基质中的回收率范围为83.2%~103.2%,相对标准偏差(RSD, *n*=6)范围为1.4%~5.6%。采用该方法对不同基质类型的化妆品样品进行筛查,共发现5批阳性样品,其中氯倍他索乙酸酯的含量范围为1.1~48.1 μg/g。该方法操作简单,灵敏可靠,适用于不同基质类型化妆品的高通量定性定量筛查分析,为监测化妆品中的非法添加提供了技术支持及理论依据。

## Abstract (en)

At present, new prohibited substances are becoming more common illegal additions in cosmetics. Clobetasol acetate is a new glucocorticoid, which is not covered in the current national standards and is a homologue of clobetasol propionate. A method was established for the determination of clobetasol acetate as a new glucocorticoid (GC) in cosmetics by ultra performance liquid chromatography-tandem mass spectrometry (UPLC-MS/MS). Five common cosmetic matrices were suitable for this new method: creams, gels, clay masks, masks and lotions. Four pretreatment methods were compared: direct extraction by acetonitrile, PRiME pass-through column purification, solid-phase extraction (SPE) purification, and QuEChERS purification. Further, the effects of different extraction efficiencies of the target compound, such as extraction solvents and extraction time, were investigated. The MS parameters, such as ion mode, cone voltage and collision energy of ion pairs of the target compound, were optimized. The chromatographic separation conditions and response intensities of the target compound in different mobile phases were compared. Based on the experimental results, the optimal extraction method was determined to be direct extraction, wherein the samples were vortex dispersed with acetonitrile, ultrasonic extraction over 30 min and filtered by a 0.22 μm organic millipore filter, and then the samples were detected by UPLC-MS/MS. The concentrated extracts were separated on a Waters CORTECS C_18_ column (150 mm×2.1 mm, 2.7 μm), where the water and acetonitrile were used as the mobile phases for gradient elution. The target compound was detected with the multiple reaction monitoring (MRM) mode under electrospray ionization and positive ion scanning (ESI^+^). Quantitative analysis was performed by matrix matching standard curve. Under the optimum conditions, the target compound had good linear fitting in the range of 0.9-37 μg/L. The linear correlation coefficient (*R*^2^) was greater than 0.99, the limit of quantification (LOQ) of the method was 0.09 μg/g and the limit of detection (LOD) was 0.03 μg/g for these five different cosmetic matrices. The recovery test was conducted under three spiked levels: 1, 2 and 10 times of LOQ. The recoveries of the tested substance were between 83.2% and 103.2% in these five cosmetic matrices, and the relative standard deviations (RSDs, *n*=6) were between 1.4% and 5.6%. This method was used to screen cosmetic samples of different matrix types, and a total of five positive samples were found, in which the content range of clobetasol acetate was from 1.1 to 48.1 μg/g. In conclusion, the method is simple, sensitive and reliable, and is suitable for high-throughput qualitative and quantitative screening, and the analysis of cosmetics with different matrix types. Moreover, the method provides crucial technical support and a theoretical basis for the establishment of feasible detection standards for clobetasol acetate in China, as well as for the control of the compound in cosmetics. This method has important practical significance to implement management measures of illegal additions in cosmetics.

氯倍他索乙酸酯是一种新型的糖皮质激素,具有孕甾烷基本母核,同时含有1,4-二烯-3-20-二酮,与氯倍他索丙酸酯结构相似(见图1),仅17位侧链上乙酸酯取代了丙酸酯,故药理作用及不良反应相似,具有抗炎、抗过敏、抗休克和免疫抑制等药理作用^[1,2]^,同时长期使用,也易形成激素依赖性,甚至可能引发骨质疏松、高血压及糖尿病等不良反应^[3⇓-5]^。我国^[6]^以及韩国^[7]^、日本^[8]^、欧盟^[9]^、东盟^[10]^等国家和地区都将糖皮质激素列为化妆品禁用原料。目前,化妆品中糖皮质激素相关检测标准^[6,11⇓⇓-14]^、文献^[3⇓-5,15⇓⇓⇓⇓⇓⇓-22]^及相关通报^[23]^中暂未见氯倍他索乙酸酯的检测方法相关报道。然而在日常工作中,发现了疑似添加新型糖皮质激素氯倍他索乙酸酯的现象。因此开发化妆品中氯倍他索乙酸酯定性定量检测方法迫在眉睫。

**图1:**
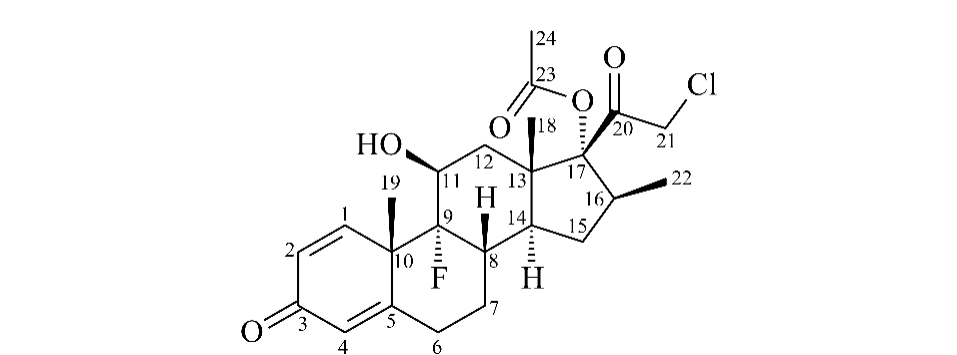
氯倍他索乙酸酯的结构

目前,化妆品中糖皮质激素的检测方法以液相色谱-高分辨质谱法(LC-HRMS)^[4,15,24]^、液相色谱-串联质谱法(LC-MS/MS)^[3,5,11⇓⇓-14,16⇓⇓⇓⇓⇓-22,25⇓⇓-28]^为主。液相色谱-高分辨质谱法有高分辨率和高质量精度的优势,可快速识别、定量和鉴定化合物^[29]^,但操作成本高、数据分析难度大,相关方法的标准化和推广应用还有一定难度^[30]^;液相色谱-串联质谱法(LC-MS/MS)具有特异性强、检出限低等特点,能够快速、准确测定化妆品中的糖皮质激素^[3⇓-5,16-17,20⇓-22,26⇓-28]^,是复杂基质中定性定量的首选标准方法。

本研究采用超高效液相色谱-串联质谱法(UPLC-MS/MS),针对化妆品基质复杂的特点,考察了4种前处理方法,并优化提取溶剂及色谱、质谱条件。同时针对化妆品中常见的5种基质(膏霜乳、凝胶、泥、贴膜和液体(水)),建立了新型糖皮质激素氯倍他索乙酸酯测定方法,为监督执法提供有力的技术支撑,保障广大消费者的用妆安全。

## 1 实验部分

### 1.1 仪器、试剂与材料

Waters Acquity UPLC/XEVO^TM^TQ-S液相色谱-三重四极杆质谱仪(美国Waters公司); Milli-Q超纯水器(美国Millipore公司); Fotector Plus高通量全自动固相萃取仪(中国睿科仪器有限公司); 45位N-EVAP氮吹仪(美国Organomation公司);台式离心机(德国Thermo Biofuge公司)。

甲醇和乙腈均为色谱纯,购自德国默克公司;乙酸(acetic acid)和甲酸(formic acid)均为色谱纯,购自上海Aladdin公司;氯化钠、亚铁氰化钾、乙酸锌和硫酸镁均为分析纯,购自国药集团化学试剂有限公司;3 mL/60 mg Oasis^®^ HLB固相萃取柱、3 mL/60 mg Oasis^®^ PRiME固相萃取柱均购自美国Waters公司。

### 1.2 标准溶液的配制

对照品氯倍他索乙酸酯由倍他米松合成得到。氯倍他索乙酸酯的分子式为C_24_H_30_ClFO_5_,相对分子质量为452.18。氯倍他索乙酸酯用乙腈溶解稀释配制成质量浓度为1 g/L的标准物质储备溶液,置于-20 ℃冷冻保存。使用时准确移取标准物质储备溶液适量,用乙腈稀释定容,配制成质量浓度为200 μg/L的标准中间液。

准确移取标准中间液适量,用基质空白提取液稀释定容,配制成质量浓度为1、2、4、10、20、40 μg/L的系列基质标准工作溶液。

### 1.3 样品处理

#### 1.3.1 样品前处理

称取样品0.2 g(精确至0.001 g),置于10 mL具塞比色管中,加入少量乙腈,涡旋分散均匀后,加入乙腈约8 mL,超声提取30 min,静置至室温,用乙腈定容至刻度,以4000 r/min转速离心10 min。取上清液经0.22 μm有机滤膜过滤,滤液作为供试品溶液备用(供试品溶液可根据实际浓度进行适当稀释)。

#### 1.3.2 基质空白提取液的制备

称取空白试样0.2 g(精确至0.001 g),置于10 mL具塞比色管中,按照1.3.1节操作处理,得到基质空白提取液,用于配制系列基质标准工作溶液。

### 1.4 仪器条件

#### 1.4.1 色谱条件

色谱柱:Waters Cortecs C_18_色谱柱(150 mm×2.1 mm, 2.7 μm);流动相A:水,流动相B:乙腈;柱温:35 ℃;流速:0.4 mL/min;进样量:2 μL。梯度洗脱程序:0~2 min, 40%B; 2~5 min, 40%B~95%B; 5~6.5 min, 95%B; 6.5~6.6 min, 95%B~40%B; 6.6~8 min, 40%B。

#### 1.4.2 质谱条件

电喷雾正离子扫描,多反应监测(MRM)模式,毛细管电压:3.2 kV;脱溶剂气流速:800 L/h;脱溶剂气温度:350 ℃;锥孔气流速:150 L/h。其他质谱参数见表1。

**表1:** 氯倍他索乙酸酯的保留时间和质谱参数

Compound	Retention time/ min	Parent ion (m/z)	Quantitative/ qualitative ions (m/z)	Cone voltage/ V	Collison energies/ V
Clobetasol	4.67	453.2	278.2/263.2	24	24/28
acetate					

## 2 结果与讨论

### 2.1 UPLC-MS/MS条件优化

#### 2.1.1 质谱条件的优化

在电喷雾离子源下,将100 μg/L的标准溶液注入离子源,分别采用正离子和负离子模式对目标化合物进行一级质谱全扫描。正离子模式的响应远高于负离子模式,在正离子模式下,氯倍他索乙酸酯可产生[M+H]^+^、[M+H-H_2_O]^+^、[M+H-HF]^+^准分子离子峰,[M+H]^+^准分子离子峰响应最强且稳定,故以[M+H]^+^离子(*m/z*=453.2)作为母离子并确定最佳的锥孔电压。然后进行二级质谱优化,选取响应较强的碎片作为定量和定性离子,并优化碰撞能量,经优化后得到的质谱条件参数见表1。

#### 2.1.2 色谱条件的优化

查阅标准及文献,分离糖皮质激素的流动相体系中有机相主要为乙腈,水相主要以甲酸水溶液、乙酸水溶液和水为主^[3⇓-5,31⇓⇓⇓-35]^。故选择0.1%甲酸水-乙腈、0.1%乙酸水-乙腈、水-乙腈作为流动相,考察被测物质的峰形及响应。结果显示,在3种流动相条件下,目标物都能得到良好的峰形及色谱峰响应,在水-乙腈的流动相体系条件下,色谱峰响应最大,故选择以水-乙腈作为流动相条件。

### 2.2 前处理方法的优化

#### 2.2.1 前处理方法的选择

参考标准及文献,目前化妆品中糖皮质激素类提取主要有①乙腈直接提取法^[6]^、②固相萃取法^[3]^、③PRiME通过式固相萃取法^[17]^及④QuEChERS法^[4]^等4种前处理方法,故以膏霜乳类为典型基质,采用基质匹配标准溶液考察了目标化合物在上述4种前处理方法下的提取效果,见图2。采用空白基质加标样品溶液中目标化合物峰面积/基质匹配标准溶液中目标化合物峰面积×100%的公式计算回收率。结果显示,乙腈直接提取法及PRiME通过式固相萃取法都能得到较好的回收率且回收率相近,但是直接提取法步骤简单便捷,更节约人力及成本,故选择乙腈直接提取法作为前处理方法。

**图2:**
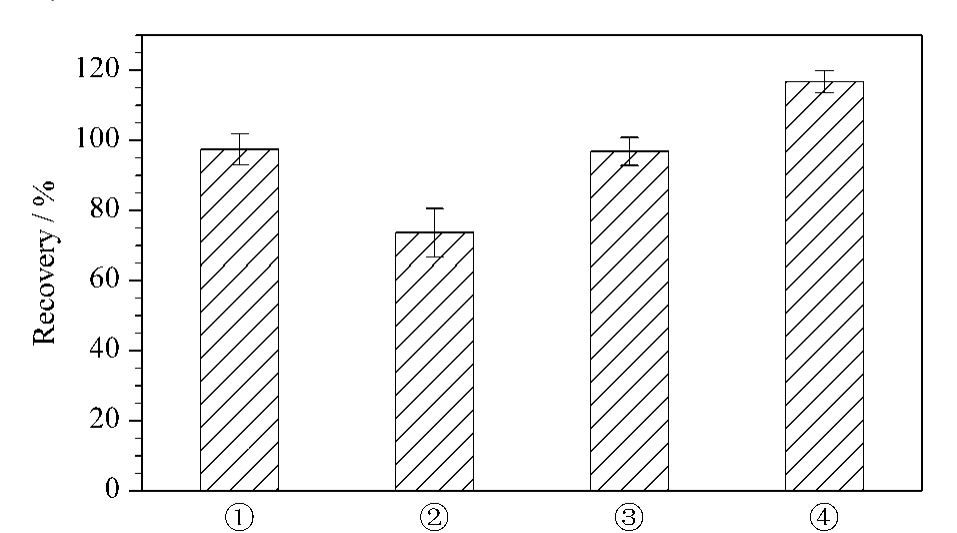
目标化合物在不同样品前处理方法下的 提取回收率(*n*=6)

#### 2.2.2 提取溶剂的选择

以膏霜乳类为典型基质,对乙腈直接提取法进行优化,分别考察了6种提取溶剂:①乙腈、②乙腈-水(70∶30, v/v)、③乙腈-水(50∶50, v/v)、④甲醇、⑤甲醇-水(70∶30, v/v)、⑥甲醇-水(50∶50, v/v)的提取效果,见图3。

**图3:**
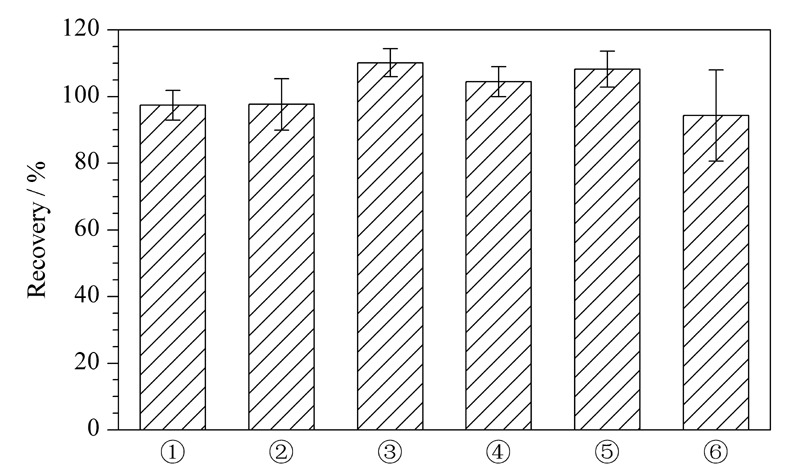
目标化合物在不同提取溶剂条件下的提取回收率(*n*=6)

结果显示,6种提取溶剂下,目标化合物均能呈现较好的峰形,且回收率相差不大。为减小溶剂效应的影响,选择色谱条件中的流动相乙腈为提取溶剂,且乙腈作为提取溶剂时,样品溶液更澄清更易过滤,更适合高通量筛查。

#### 2.2.3 超声时间的选择

以膏霜乳类为典型基质,对超声提取的时间进行考察,采用2.2.1节方法计算回收率,分别考察了在10、20和30 min 3种超声提取时间下的提取效果,见图4。结果显示,超声时间越长,提取效果越好,为保证能得到更好的回收率,选择30 min作为超声提取时间。

**图4:**
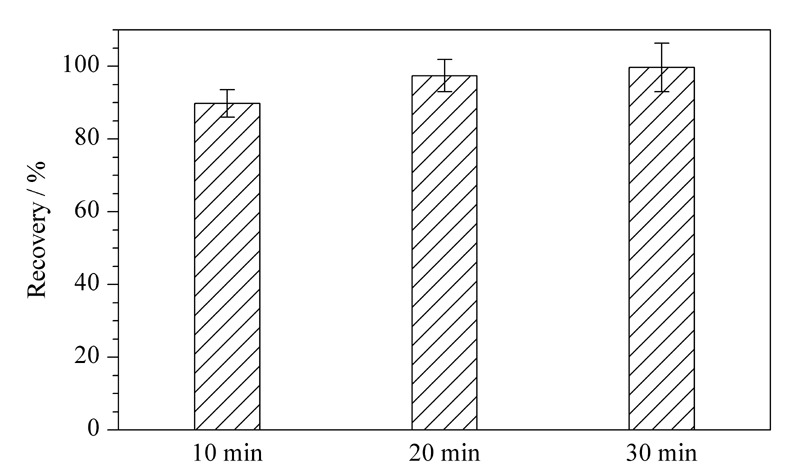
目标化合物在不同超声时间下的提取回收率(*n*=6)

#### 2.2.4 基质效应的考察

本研究采用ME=基质标准曲线的斜率/溶剂标准曲线的斜率×100%的计算方法对目标化合物的基质效应进行评价^[27]^。若ME>100%,则为基质增强效应,ME<100%,则为基质抑制效应。ME与100%的差值越大,则表明基质效应越明显^[28]^。研究考察了膏霜乳类、凝胶类、泥类、贴膜类和液体(水)5种基质类型的基质效应,见表2。结果表明,除液体(水)外,其余基质的ME值都低于80%,存在明显的基质抑制效应。为了消除基质的影响,保证分析结果的准确性,用基质空白提取液稀释标准溶液配制得到系列基质标准工作溶液,采用基质匹配标准曲线进行定量计算。

**表2:** 在不同基质类型下目标化合物的线性范围、线性方程、相关系数、检出限、定量限及基质效应

Matrix type	Linear range/(μg/L)	Linear equation	R^2^	LOD/(μg/g)	LOQ/(μg/g)	ME/%
Standard solution	0.9-37	y=2.57×10^3^x+6.39×10^2^	0.9998	0.03	0.09	/
Cream	0.9-37	y=1.34×10^3^x+5.04×10^2^	0.9982	0.03	0.09	52.2
Gel	0.9-37	y=8.73×10^2^x+2.17×10^2^	0.9958	0.03	0.09	34.0
Clay masks	0.9-37	y=1.89×10^3^x+9.13×10^2^	0.9984	0.03	0.09	73.7
Mask	0.9-37	y=1.95×10^3^x+9.02×10^2^	0.9998	0.03	0.09	76.0
Lotion	0.9-37	y=2.15×10^3^x+1.08×10^3^	0.9994	0.03	0.09	83.8

y: peak area; x: mass concentration, μg/L.

### 2.3 方法的线性关系与灵敏度

本研究针对膏霜乳、凝胶、泥、贴膜和液体(水)5种基质进行考察。采用基质空白提取液稀释标准溶液配制系列基质标准溶液,以被测化合物定量离子对的峰面积为纵坐标(*y*),以被测物质的质量浓度为横坐标(*x*, μg/L)绘制基质标准工作曲线,考察被测物的线性关系,结果见表2。5种基质,被测物质在0.9~37 μg/L的线性范围内线性拟合良好,线性相关系数(*R*^2^)均超过0.99。

### 2.4 方法的准确度和精密度

采用在膏霜乳类,凝胶类、泥类、贴膜类和液体类(水)5种基质类型的空白样品中添加定量限、2倍定量限、10倍定量限3个水平的被测物质标准溶液,记录被测物质的峰面积,代入基质标准曲线计算被测物质加标回收率和精密度(RSD, *n*=6),见表3。氯倍他索乙酸酯在5种基质中的平均回收率范围为83.2%~103.2%,精密度范围为1.4%~5.6%。

**表3:** 不同基质类型下目标化合物的加标回收率和精密度(n=6)

Compound	Matrix type	0.09 μg/g			0.19 μg/g			0.94 μg/g	
		Recovery/%	RSD/%		Recovery/%	RSD/%		Recovery/%	RSD/%
Clobetasol	cream	94.4	3.3		96.2	2.2		101.1	5.6
acetate	gel	85.5	2.4		100.4	3.8		95.6	4.4
	clay mask	94.0	5.3		91.9	4.4		92.4	1.4
	mask	91.3	3.8		100.1	3.9		103.2	3.6
	lotion	83.2	3.9		94.8	3.2		96.5	1.4

### 2.5 方法的特异性

采用空白溶剂、标准溶液(1.9 μg/L)、5种不同基质空白样品及空白样品添加标准溶液(1.9 μg/L)分别进行测定。在MRM模式下同时监测2个离子对,通过保留时间及离子丰度比进行定性,确保结果特异性。

氯倍他索乙酸酯的保留时间为4.67 min,空白溶剂及5种空白基质在4.67 min处均没有色谱峰。可见,空白溶剂及各类别空白基质对被测目标化合物无干扰,该方法特异性良好。

### 2.6 实际样品测定

采用本法对32批不同基质类型的样品进行筛查,共有5批样品检出氯倍他索乙酸酯,检出量由小到大依次为:1.1、1.1、34.5、39.2和48.1 μg/g。上述5批样品基质类型为贴膜和凝胶,样品来源于3个不同的生产企业,抽样地点来自于生产企业、网购和小商店,见表4。这表明,目前化妆品市场上非法添加氯倍他索乙酸酯这种新型糖皮质激素的现象已经很普遍,并非个例。使用本方法可以准确地发现并测定此非法添加物,为化妆品监管提供技术支撑。典型阳性样品及对照溶液中氯倍他索乙酸酯的色谱图见图5。

**表4:** 阳性样品的样品信息和含量

No.	Matrix type	Manufacturer	Sampling site	Content/ (μg/g)
1	mask	manufacturer 1	small shop	1.1
2	mask	manufacturer 1	small shop	1.1
3	mask	manufacturer 2	small shop	34.5
4	gel	manufacturer 3	online shopping	39.2
5	gel	manufacturer 3	production unit	48.1

**图5:**
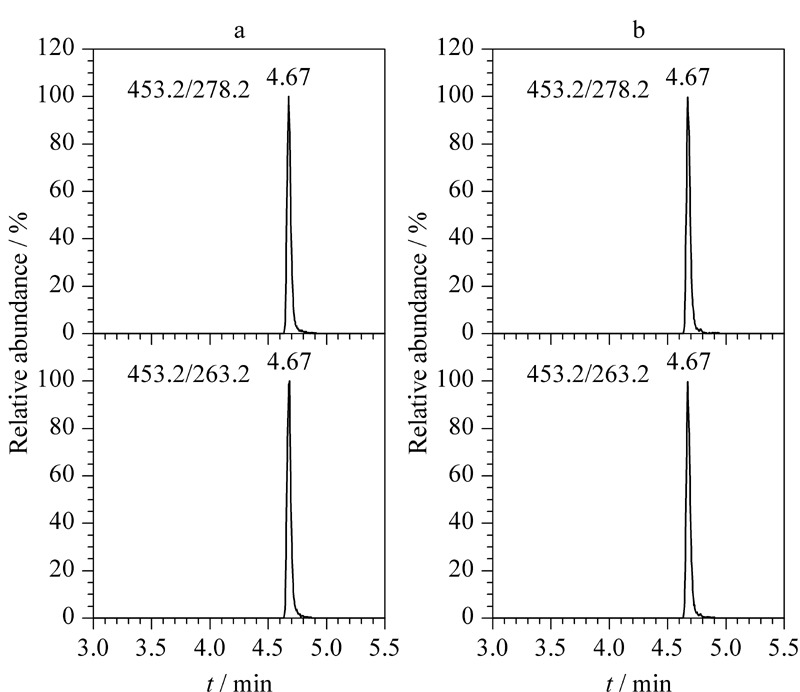
(a)典型阳性样品和(b)对照溶液中氯倍他索乙酸酯 的色谱图

## 3 结论

本研究建立了UPLC-MS/MS测定化妆品中氯倍他索乙酸酯的检测方法。氯倍他索乙酸酯作为一种新型的糖皮质激素,非法添加在化妆品中,亦会危害消费者的健康。本文采用乙腈直接提取法对样品进行前处理,基质标准曲线进行准确定量,方法简单高效,适用于化妆品中常见的膏霜乳、凝胶、泥、贴膜和液体(水)5种基质类型化妆品中氯倍他索乙酸酯的高通量筛查以及准确定性定量。该研究结果可为化妆品的日常监管提供技术及数据支持。

## References

[1] Yang B F, Chen J G. Pharmacology. 9th ed. Beijing: People’s Medical Publishing House, 2018

[2] You Q D. Medicinal Chemistry. 8th ed. Beijing: People’s Medical Publishing House, 2016

[3] Yang P P, Liu H, Li L X. China Surfactant Detergent & Cosmetics, 2021, 51(5): 468

[4] Luo H T, Huang X L, Wu H Q, et al. Chinese Journal of Analytical Chemistry, 2017, 45(9): 1381

[5] Qiao Y S, Dong Y L, Huang C F, et al. China Surfactant Detergent & Cosmetics, 2021, 51(12): 1259

[6] China Food and Drug Administration. Safety and Technical Standards for Cosmetics (2015 Edition).(2015-12-23) [2022-03-14]. https://www.nmpa.gov.cn/directory/web/nmpa/images/MjAxNcTqtdoyNji6xbmruOa4vbz+LnBkZg==.pdfhttps://www.nmpa.gov.cn/directory/web/nmpa/images/MjAxNcTqtdoyNji6xbmruOa4vbz+LnBkZg==.pdf

[7] Korea Cosmetics Regulation. General Administration of Quality Supervision, Inspection and Quarantine of the People’s Republic of China, transl. Beijing: China Metrology Press, 2013

[8] Japan Cosmetics Regulation. General Administration of Quality Supervision, Inspection and Quarantine of the People’s Republic of China, transl. Beijing: China Metrology Press, 2011

[9] EC) No 1223/2009

[10] Association of Southeast Asian Nations ASEAN Cosmetics Regulations. General Administration of Quality Supervision, Inspection and Quarantine of the People’s Republic of China, transl. Beijing: China Metrology Press, 2010

[11] GB/T 24800.2-2009

[12] GB/T 40145-2021

[13] SN/T 2533-2010

[14] SN/T 4504-2016

[15] Li Y J, Zhao X L, Cen L Y, et al. Flavour Fragrance Cosmetics, 2018(1): 58

[16] Luo H T, Huang X L, Wu H Q, et al. Chinese Journal of Chromatography, 2017, 35(8): 816 29048815 10.3724/SP.J.1123.2017.04005

[17] Liu H, Yang P P, Li L X. Journal of Instrumental Analysis, 2020, 39(9): 1112

[18] Liu H, Zhang J Y, Hu B, et al. Flavour Fragrance Cosmetics, 2020(4): 83

[19] Weng D H, Liu J, Jiang Q Q, et al. Flavour Fragrance Cosmetics, 2019(3): 45

[20] Pan X H, Yin S, Liu Y L, et al. Chinese Journal of Chromatography, 2018, 36(4): 356 30136518 10.3724/SP.J.1123.2017.10035

[21] Dong Y L, Liu Z, Wang H Y, et al. China Surfactant Detergent & Cosmetics, 2020, 50(12): 885

[22] Yang G Y, Sha L N, Liang Q Y, et al. Chinese Journal of Analysis Laboratory, 2021, 40(8): 959

[23] Zhangzhou Health Commission. Notification on the Progress of Investigation and Disposal of “Ouai Antibacterial Cream” Incident. (2021-01-17) [2022-03-10]. http://wjw.zhangzhou.gov.cn/cms/html/zzswshjhsywyh/2021-01-17/1663659826.htmlhttp://wjw.zhangzhou.gov.cn/cms/html/zzswshjhsywyh/2021-01-17/1663659826.html

[24] Meng X S, Bai H, Guo T, et al. J Chromatogr A, 2017, 1528: 61 29122284 10.1016/j.chroma.2017.11.004

[25] Jian L H, Yuan X Q, Han J, et al. Rapid Commun Mass Spectrom, 2021, 35(3): e8999 33140453 10.1002/rcm.8999

[26] Zhan J, Ni M L, Zhao H Y, et al. J Sep Sci, 2014, 37(24): 3684 25311438 10.1002/jssc.201400698

[27] Jelena B, GoluboviĆ B M O A, ZeĆeviĆ M L. J Sep Sci, 2014, 37(24): 3684 25311438

[28] Jessica F, Vincenza A. J Pharm Biomed Anal, 2014, 91: 185 24463045

[29] He M L, Guo C C, Shi F, et al. Chinese Journal of Pharmaceutical Analysis, 2019, 39(1): 105

[30] Wang B F, Xu L, Xu Z Z, et al. Food Science, 2021, 42(7): 301

[31] Xu D M, Zeng S M, Liu X C, et al. Chinese Journal of Chromatography, 2022, 40(5): 423 35478001 10.3724/SP.J.1123.2021.08008 PMC9404142

[32] Zhou Y S, Gong J, Yang K X, et al. Chinese Journal of Chromatography, 2022, 40(2): 165 35080163 10.3724/SP.J.1123.2021.03025 PMC9404236

[33] Yao Q, Guo Y, Xue J, et al. J Pharm Biomed Anal, 2020, 179: 112980 31744668 10.1016/j.jpba.2019.112980

[34] Liu M, Li X, Li J, et al. J Colloid Interface Sci, 2017, 504: 124 28535412 10.1016/j.jcis.2017.05.041

[35] Giaccone V, Macaluso A, Cammilleri G, et al. Food Add Contam B, 2018, 11(3): 201 10.1080/19393210.2018.1470578 29716443

